# Estimating the Impact of State Budget Cuts and Redirection of Prevention Resources on the HIV Epidemic in 59 California Local Health Departments

**DOI:** 10.1371/journal.pone.0055713

**Published:** 2013-03-08

**Authors:** Feng Lin, Arielle Lasry, Stephanie L. Sansom, Richard J. Wolitski

**Affiliations:** Division of HIV/AIDS Prevention, National Center for HIV/AIDS, Viral Hepatitis, STD, and TB Prevention, Centers for Disease Control and Prevention, Atlanta, Georgia, United States of America; Public Health Agency of Barcelona, Spain

## Abstract

**Background:**

In the wake of a national economic downturn, the state of California, in 2009–2010, implemented budget cuts that eliminated state funding of HIV prevention and testing. To mitigate the effect of these cuts remaining federal funds were redirected. This analysis estimates the impact of these budget cuts and reallocation of resources on HIV transmission and associated HIV treatment costs.

**Methods and Findings:**

We estimated the effect of the budget cuts and reallocation for California county health departments (excluding Los Angeles and San Francisco) on the number of individuals living with or at-risk for HIV who received HIV prevention services. We used a Bernoulli model to estimate the number of new infections that would occur each year as a result of the changes, and assigned lifetime treatment costs to those new infections. We explored the effect of redirecting federal funds to more cost-effective programs, as well as the potential effect of allocating funds proportionately by transmission category. We estimated that cutting HIV prevention resulted in 55 new infections that were associated with $20 million in lifetime treatment costs. The redirection of federal funds to more cost-effective programs averted 15 HIV infections. If HIV prevention funding were allocated proportionately to transmission categories, we estimated that HIV infections could be reduced below the number that occurred annually before the state budget cuts.

**Conclusions:**

Reducing funding for HIV prevention may result in short-term savings at the expense of additional HIV infections and increased HIV treatment costs. Existing HIV prevention funds would likely have a greater impact on the epidemic if they were allocated to the more cost-effective programs and the populations most likely to acquire and transmit the infection.

## Introduction

The HIV epidemic continues to be a major public health problem in the United States. Nearly 1.2 million persons are living with the disease [1], and about 50,000 new infections occur annually [2]. In 2010, the White House issued the National HIV/AIDS Strategy, setting goals for decreasing the annual number of new infections by 25% by 2015, increasing access to care and improving the health of persons living with HIV, and reducing HIV-related disparities [3]. Achieving those goals will require sufficient funding for HIV prevention and treatment, and more strategic use of existing funding.

In the United States, HIV prevention programs are primarily funded by the federal, state and local governments and are administered by state and local health departments. In fiscal year 2007, 58% of prevention funding ($337 million) was provided by the federal government, 35% ($205 million) by state and local governments, and 7% ($39 million) by non-governmental entities, such as foundations and pharmaceutical and diagnostic companies [4]. Although there have been modest increases in federal HIV prevention funding since 2007 [5], state governments have experienced deficits during that period that have resulted in reductions in or eliminations of a wide range of programs, including HIV prevention [6]. As of mid-2010, of the 33 states funding HIV prevention [5], eight had reported cuts to HIV testing programs, nine to behavioral interventions, such as those aimed at risk reduction, and seven to partner services [6].

In 2009, an estimated 107,138 persons were living with HIV in California, and 4,981 were newly diagnosed with HIV. Men who have sex with men (MSM) accounted for 73% of HIV prevalence and almost 80% of new diagnoses. Historically, the state of California has allocated a substantial amount of state funds to HIV prevention [4], [7], [8]. In 2005–2007, the state provided 75% ($41 million) of the combined state and federal funding for HIV prevention, while the federal government provided the remaining 25% ($14 million) [7], [8]. However, since fiscal year 2009–2010, the state health department has relied solely on federal funding to support HIV prevention programs [7], [8]. Arnold et al. used a qualitative approach to examine the impact of the California state budget cuts on the provision and access of HIV-related services in Alameda, Fresno, and Los Angeles Counties [8]. In this paper, we quantify the effect of the reduced prevention budget on new HIV infections and their associated treatment costs, and explore the effects of different allocation strategies.

## Methods

### Geographical Scope of Analysis

Historically, HIV prevention funds in California were allocated to 58 county and 3 city health departments (Berkeley, Long Beach, and Pasadena), for a total of 61 local health jurisdictions. However, complete data were not available for Los Angeles and San Francisco which were thus excluded from this analysis. For the years preceding the budget cut, or what we refer to as the “pre-cut” timeframe, we considered 59 local health jurisdictions, including 56 counties and the 3 cities. In the “post-cut” timeframe, we considered the subset of the 59 local health jurisdictions that continued to receive state-administered HIV prevention funds, again excluding Los Angeles and San Francisco.

### Input Data

The California Department of Public Health’s Office of AIDS provided annual epidemiologic, budgetary and program data for the state’s fiscal years 2005–2006 through 2009–2010. Data included HIV prevalence and the annual number of new HIV cases diagnosed, by transmission category, including MSM, injecting drug users (IDU) and high-risk heterosexuals (HET), defined as heterosexual contact with a person known to have, or to be at high risk for, HIV infection [9]. Data on state and federal expenditures on programs and persons served by each program also included a breakdown by HIV serostatus and transmission category. In addition, the data included the number of counties and cities funded for HIV prevention and the number of HIV prevention agencies in those jurisdictions that received state and federal HIV prevention funds administered by the Office before and after the state budget cuts.

We used a Bernoulli model to estimate, by transmission category, the annual rate at which HIV-infected individuals transmit the disease to uninfected persons and the annual risk of infection for uninfected persons [10]. The inputs to the Bernoulli model included type of sex act, annual number of sex acts, proportion of all sex acts protected by condom use, annual number of partners, number of contaminated needle sharing acts, per-act HIV transmission probabilities, and the effectiveness of condoms and antiretroviral treatment (ART) in reducing per-act transmission probabilities. The inputs also included effects on transmission rates associated with HIV testing and risk reduction programs. Details about sexual and drug injecting behaviors included in the model are reported in Table 1. We calculated annual transmission rates, with and without intervention effects, for male and female HET and IDU as well as for MSM. With these rates, we were able to estimate the number of new infections associated with the state HIV prevention budget cuts.

**Table 1:** Summary of key model parameters.

Parameter	Value (bounds for sensitivity analyses)	Source
HIV prevalence in California		
HET	1.0% (0.75–1.25%)	[33]
IDU	5.9% (4.0–10.2%)	[34]
MSM	19.1% (12.8–25.35%)	[35]
Per-act HIV transmission probability^1^		
Vaginal receptive	0.08% (0.06–0.11%)	[36]
Vaginal insertive	0.04% (0.01–0.14%)	[36]
Anal receptive	1.4% (0.2–2.5%)	[37]
Anal insertive	0.7% (0%–1.3%)	[37]
Contaminated needle sharing	0.30% (0.24%–0.65%)	[38]
Annual number of sexual partners		
HET	1.21 (1–20)	[39]
IDU	3 (1–20)	[40]
MSM	3.5 (1–20)	[41]
Annual number of sex acts all partners^2^		
HET	70 (26–365)	[42]–[44]
IDU	70 (26–365)	[42]–[44]
MSM	70 (26–365)	[42]–[44]
Annual number of intravenous injections (all partners)	200 (100–300)	[45], [46]
Proportion of protected sex acts for undiagnosed HIV-infected individuals and uninfected individuals who do not participate in risk reduction		
HET	20% (10–50%)	[47]
IDU	20% (10–50%)	[47]
MSM	55% (40–80%)	[48]
Proportion of needle sharing acts among all injections for IDU who do not receive anyprevention interventions	15% (5–25%)	[45], [46]
Proportion of new diagnoses among positives notified of test results	60% (40–80%)	[25], [26]
Reduction in unprotected sex for aware HIV-infected individuals compared with unaware	53% (45–60%)	[11]
Intervention effect size of HIV education and risk reduction for positive clients^3^	27% (0–40%)	[16]–[20]
Intervention effect size of HIV education and risk reduction for negative clients^3^	12% (0–20%)	[21]–[23]
Condom efficacy for per-act transmission	80% (65–95%)	[49]
Reduction in per-act transmissibility for HIV-infected individuals who achieve viralload suppression	96% (50–100%)	[50]–[54]
Reduction in needle sharing infectivity for infected IDU who achieve viral load suppression	50% (0–90%)	[55]
Proportion of diagnosed HIV-infected persons who are linked to care	77% (60–85%)	[14]
Proportion of HIV-infected persons linked to care who are retained in care	66% (50–80%)	[14]
Proportion of HIV-infected persons, retained in care, who have started ART	88% (70–90%)	[14]
Proportion of HIV-infected persons who started ART who are adherent to ART (i.e., achieveviral load suppression)	77% (60–85%)	[14]
HIV lifetime treatment cost (2009 $)	367,134 (184,000–550,000)	[24]
Proportion of protected sex acts for HIV-positive aware w/o participating in risk reduction^4^		
HET	73%	Calculated
IDU	73%	Calculated
MSM	85%	Calculated

1 In the Bernoulli model, we assumed HET and IDU females engaged in vaginal receptive sex, while HET and IDU males engaged in vaginal insertive sex. We also considered transmission via contaminated needle sharing for IDU. For MSM, we assumed 50% of their sex acts were insertive anal and 50% were receptive anal.

2 We assumed every individual in a particular transmission category had the same number of annual sex acts. The annual number of sex acts for HET was reported in the National Survey of Family Growth (NSFG) and the National Survey of Sexual Health and Behavior (NSSHB) for HET. We assumed IDU and MSM had the same annual number of sex acts as HET.

3 The effect sizes of risk reduction for HIV-infected and uninfected at-risk individuals were estimated by the percent reduction in unprotected sex acts (unprotected vaginal sex or anal sex). We included behavioral studies that reported the reduction in number (or percent) of unprotected sex acts. We took the median values of the reviewed studies in which the reported reduction in unprotected sex acts between intervention and control groups was statistically significant.

4 The proportion of protected sex acts among HIV-positive aware persons who do not receive risk reduction was calculated from the proportion of protected sex for unaware HIV positive persons and the reduction in unprotected sex for aware HIV-infected persons. That is, the proportion of protected sex acts for aware HIV-infected persons = 1-(1- proportion of protected sex acts for unaware HIV-positive persons)×(1- reduction in unprotected sex acts for aware HIV-infected persons).

We considered testing and partner services, and HIV education and risk reduction in our analysis because reasonably robust data exist on their efficacy and the programmatic data on their service provision were complete. HIV testing and partner services diagnose and notify HIV-infected individuals of their infection. HIV-infected individuals, once diagnosed, have been found to reduce the proportion of their sex acts that are unprotected by condoms by 53% [11]–[13]. In addition, a proportion of HIV-diagnosed individuals will achieve viral load suppression after receiving ART [14], greatly reducing their per-act HIV transmission probabilities [15]. We assumed testing and partner services conferred no benefits to uninfected individuals. HIV education and risk reduction services for HIV-infected individuals have been estimated to reduce the proportion of a participant’s sex acts that are unprotected by condoms by 27% [16]–[20] (in addition to the 53% reduction associated with new diagnosis); risk reduction for HIV-uninfected individuals has been estimated to reduce the proportion by 12% [21]–[23].

In California, the state HIV prevention budget reductions started in fiscal year 2008–2009, and were followed by a more drastic cut in fiscal year 2009–2010. In fiscal year 2008–2009, $4.6 million in temporary state funding ended for HIV prevention in low-prevalence jurisdictions. This cut was followed in fiscal year 2009–2010 by the elimination of the rest of the $11.4 million state funding for HIV prevention. To explore the impact of the combined state budget cuts on HIV prevention, we defined fiscal year 2009–2010 as our post-cut timeframe, and compared it with the years before any of the state budget cuts began: fiscal year 2005–2006 through 2007–2008. To get a stable and robust representation of the budget and services before any cuts occurred, we averaged the values from fiscal year 2005–2006 through 2007–2008. We referred to this as the pre-cut timeframe.

### Base Case

In the base case analysis, we compared the pre-cut and post-cut budget and allocation strategies during each timeframe with respect to the number of individuals served, the reported number of diagnoses, and the estimated number of additional HIV cases compared with HIV cases during the pre-cut timeframe in the 59 jurisdictions. We applied lifetime HIV treatment costs to the additional cases of HIV to estimate the financial impact of the budget cuts. We used an HIV lifetime treatment cost of $367,134, in 2009 U.S. dollars [24], discounted by 3% to the time of infection.

### Analytic Scenarios

To understand the potential impact of different budget allocation strategies we assessed three additional hypothetical scenarios. First, using the 2009–2010 budget, we compared outcomes under the actual 2009–2010 budget allocation to testing and risk reduction programs (that is, the proportion of total funding allocated to testing compared with risk reduction programs), to those that would have been expected had the same amount of funding instead been distributed according to the pre-cut allocation to testing and risk reduction programs. In the second scenario, we applied the 2009–2010 budget and the 2009–2010 allocation to testing and risk reduction programs, and within each program type, we allocated services to each transmission category proportionate to that group’s contribution to all living cases of HIV in the funded jurisdictions. For instance, if MSM comprised 75% of all those infected with HIV in the areas under consideration, we allocated 75% of services to MSM. In the third scenario, we allocated the entire pre-cut HIV prevention budget to the 59 original jurisdictions, but this time we allocated to programs under the post-cut allocation, and we allocated to transmission categories proportionate to each group’s contribution to all living cases of HIV in the 59 jurisdictions.

In each scenario, to determine the number of tests performed and clients served by risk reduction programs, we divided the total amount allocated to each prevention program by the cost per person tested or client served, as reported by the state for fiscal year 2009–2010. To determine the number of positive test results, we multiplied the number tested by the HIV sero-positive rate by transmission category reported among those tested in 2009–2010. To estimate the number of new diagnoses, we multiplied the number of positive tests by 60% [25], [26]. For each scenario, we estimated the difference in the number of new infections expected annually compared to the reported annual number of new diagnoses among the 59 jurisdictions in the pre-cut timeframe.

### Sensitivity Analysis

We performed both one-way and probabilistic sensitivity analyses on a variety of parameters, including HIV prevalence in California among the three transmission categories, the per-act transmission probabilities, the self-reported sexual behaviors that inform the Bernoulli model, and the effect of behavioral and biomedical interventions on transmission probabilities. We tested the impact of each parameter on the main outcome, the estimated number of new infections associated with the budget cuts. The probabilistic analysis provided 95% confidence intervals around each estimate of new HIV infections associated with our base case and analytic scenarios. We applied the point estimates and confidence intervals to estimates of lifetime HIV treatment costs to determine the range of costs associated with each scenario. Distributions applied to each parameter in the probabilistic analysis are described in Table S1.

## Results

During the pre-cut timeframe, the HIV prevention budget for the 59 jurisdictions was on average $21.8 million, 91% provided by the state and 9% provided by the federal government (Table 2). In those 59 jurisdictions, 51,745 persons, on average, were living with HIV/AIDS and 2,874 new HIV cases were diagnosed annually. MSM accounted for 73% of the prevalence, while IDU and HET each accounted for 13%. Among the new diagnoses, MSM, HET, and IDU accounted for 72%, 17%, and 12%, respectively. During the post-cut year, the budget was funded entirely by the federal government at $5.9 million. The $5.9 million was allocated to 15 jurisdictions that contained 87% of the HIV prevalence in all of the 59 jurisdictions [27] and an annual number of 2,470 new diagnoses (86% of the new diagnoses in all 59 jurisdictions). Among those infected in the 15 post-cut jurisdictions, MSM accounted for 74% of the prevalence, while HET and IDU each accounted for 13%. Among the new diagnoses, MSM, HET, and IDU accounted for 72%, 17%, and 11%, respectively.

**Table 2:** Summary of HIV prevention budget, services and providers funded to selected jurisdictions by the California Office of AIDS (excluding Los Angeles and San Francisco).

	Pre-cut*:Fiscal year 2005–2006 to Fiscal year 2007–2008	Post-cut: Fiscal year 2009–2010		
		Program data	Change from pre-cut** (%)	
Total prevention budget ($)	21,849,923	5,860,723	−15,989,200	(−73)
Federal funding	1,923,529	5,860,723	3,937,194	(205)
State funding	19,926,394	0	−19,926,394	(−100)
Funded prevention agencies	143#	36	−107	(−75)
Funded local health jurisdictions	59	15	−44	(−75)
HIV prevalence:	54,635&	59,908	–	–
15 jurisdictions funded in FY0910	47,328&	51,959	–	–
44 jurisdictions not funded in FY0910	7,307&	7,949	–	–
***Testing and partner services***				
Budget ($)	4,024,634	3,160,148	−864,486	(−21)
Federal funding	479,574	3,160,148	2,680,574	(559)
State funding	3,545,060	0	−3,545,060	(−100)
Number of persons served	85,636	53,545	−32,091	(−37)
Number of tests performed	83,968	53,001	−30,967	(−37)
HET	59,567	35,976	−23,591	(−40)
IDU	7,586	4,216	−3,371	(−44)
MSM	16,815	12,809	−4,006	(−24)
Number of HIV-positive clients notified of test result	813	465	−348	(−43)
HET	254	117	−138	(−54)
IDU	46	18	−28	(−62)
MSM	512	331	−182	(−35)
Sero-positive rate: all transmission categories	0.97%	0.88%	–	–
HET	0.43%	0.32%	–	–
IDU	0.61%	0.42%	–	–
MSM	3.05%	2.58%	–	–
***HIV education and risk reduction***				
Budget ($)	17,825,289	2,700,575	−15,124,714	(−85)
Federal funding	1,443,956	2,700,575	1,256,619	(87)
State funding	16,381,333	$0	−16,381,333	(−100)
Number of unique positive clients served	2,884	1,100	−1,784	(−62)
HET	1,362	560	−802	(−59)
IDU	135	45	−90	(−66)
MSM	1,387	494	−893	(−64)
Number of unique negative clients served	8,900	2,286	−6,614	(−74)
HET	5,765	1,459	−4,306	(−75)
IDU	1,090	245	−845	(−77)
MSM	2,045	582	−1,463	(−72)

* We assumed average values from fiscal year 2005–2006 to fiscal year 2007–2008 for the pre-cut scenario.

** Estimated.

# Number of funded local prevention agencies was only available in fiscal year 2007–2008.

& HIV prevalence for selected jurisdictions in California in fiscal year 2007–2008.

During the pre-cut years, 143 agencies received HIV prevention funds; afterwards, 36 agencies received funds. During the pre-cut years, more than 75% of the budget was allocated to risk reduction programs. Afterwards, about 50% went to risk reduction and 50% to testing.

During the pre-cut years, 83,968 tests were performed and an estimated 813 persons (sero-positive rate of 0.97%) were notified of a positive HIV diagnosis annually. Afterwards, the number of tests performed dropped to 53,001, and 465 persons (sero-positive rate of 0.88%) were notified of a positive HIV diagnosis. Seventy-one percent of the tests were provided to HET, 9% to IDU, and 20% to MSM in the pre-cut timeframe; post-cut, 68% of tests were provided to HET, 8% to IDU, and 24% to MSM. Among the positives notified of test results, 63% were MSM, 31% were HET and 6% were IDU in the pre-cut timeframe; post-cut, 71% were MSM, 25% were HET and 4% were IDU.

An average of 11,784 unique clients was served by risk reduction programs annually in pre-cut timeframe, including 2,884 (24%) positive clients and 8,900 (76%) negative clients. Post-cut, the number of unique risk reduction clients decreased to 3,386, including 1,100 (32%) positive clients and 2,286 (68%) negative clients. During the pre-cut years, 47% of HIV-positive risk reduction clients were HET, 5% were IDU, and 48% were MSM; while 65% of HIV-negative clients were HET, 12% were IDU, and 23% were MSM. Those proportions remained the about same following the cuts.

Based on Bernoulli models, we were able to estimate the effect of HIV prevention interventions on annual transmission and acquisition rates among MSM, HET and IDU (Table 3). We estimated that HIV-infected MSM experienced the greatest decrease (0.17) in their transmission rate following a new diagnosis of HIV, and that other transmission categories experienced an annual decrease ranging from 0.014 among HET females to 0.058 among IDU males. The annual transmission-rate decrease following risk reduction for HIV-infected persons ranged from 0.002 among IDU and HET females, to 0.025 among MSM. The annual infection-rate decrease following risk reduction for HIV negative persons ranged from 0.00002 for HET males to 0.005 for MSM.

**Table 3:** Estimates of the HIV annual transmission rate for HIV-infected individuals, the risk of infection for uninfected individuals, and the effectiveness achieved by HIV prevention activities.

	Without intervention	With intervention	Effectiveness: reduction in transmission rate or risk of infection (%)	
Testing and partner services				
Transmission rate	Unaware infected individuals	Aware infected individuals		
HET Male	0.04569	0.01838	0.02731	(60)
HET Female	0.02306	0.00924	0.01382	(60)
IDU Male	0.12619	0.06830	0.05789	(46)
IDU Female	0.10495	0.05972	0.04524	(43)
MSM	0.31589	0.14230	0.17359	(55)
HIV education and risk reduction for HIV-positive clients				
Transmission rate	Aware infected individuals who had not received risk reduction services	Aware infected individuals who had received risk reduction services		
HET Male	0.02746	0.02305	0.00441	(16)
HET Female	0.01380	0.01158	0.00223	(16)
IDU Male	0.08736	0.08321	0.00415	(5)
IDU Female	0.07454	0.07245	0.00208	(3)
MSM	0.21243	0.18704	0.02540	(12)
HIV education and risk reduction for HIV-negative clients				
Risk of infection	Uninfected individuals who had notreceived risk reduction services	Uninfected individuals who had receivedrisk reduction services		
HET Male	0.00046	0.00042	0.00004	(9)
HET Female	0.00023	0.00021	0.00002	(9)
IDU Male	0.00789	0.00765	0.00024	(3)
IDU Female	0.00657	0.00644	0.00012	(2)
MSM	0.07261	0.06745	0.00517	(7)

Based on these calculations of annual transmission and incidence rates, we estimated that 55 additional HIV infections would occur in connection with the first year of the state’s budget cut (Table 4). This represented a 1. 91% increase over the 2,874 infections otherwise expected to incur in the 59 jurisdictions at a societal cost of $20.2 million, compared to the $15.9 million reduction in funding.

**Table 4:** Comparison of budget allocations: pre-cut allocation versus actual allocation in FY0910.

	Base case: post-cut allocation, actual allocation in FY0910	Analytic allocation scenarios		
		Scenario 1: FY0910 budget, pre-cut programmatic allocation, and services to transmission categories proportionate to each group’s pre-cut allocation	Scenario 2: FY0910 budget, FY0910 programmatic allocation, and services to transmission categories proportionate to each group’s contribution to HIV prevalence	Scenario 3: Pre-cut budget, FY0910 programmatic allocation, and services to transmission categories proportionate to each group’s contribution to HIV prevalence
Total prevention budget ($)	5,860,723 (100%)	5,860,723 (100%)	5,860,723 (100%)	21,849,923 (100%)
Testing and partner services	3,160,148 (54%)	1,079,512 (18%)	3,160,148 (54%)	11,781,651 (54%)
HIV education and risk reduction	2,700,575 (46%)	4,781,211 (82%)	2,700,575 (46%)	10,068,273 (46%)
Number of local health jurisdictions funded	15	59	15	59
Number of tests performed	53,001	18,105	53,001	197,888
HET (% of 993,600 at risk HET)	35,976 (3.6%)	12,844 (1.3%)	6,879 (0.7%)	26,864 (2.7%)
IDU (% of 178,678 at risk IDU)	4,216 (2.4%)	1,636 (0.9%)	6,868 (3.8%)	26,020 (14.6%)
MSM (% of 401,592 at risk MSM)	12,809 (3.2%)	3,626 (0.9%)	39,253 (9.8%)	145,005 (36%)
Number of positives notified of test result	465	175	1,267	4,664
HET	117	55	29	112
IDU	18	10	42	166
MSM	331	110	1,196	4,386
Number of new diagnoses (60% new diagnosis rate [25], [26])	279	105	760	2,798
HET	70	33	18	67
IDU	11	6	25	100
MSM	198	66	717	2,631
Number of unique risk reduction clients	3,386	5,995	3,386	2,798
HIV-positive clients	1,100	1,467	1,100	4,101
HET	560	693	146	544
IDU	45	69	140	569
MSM	494	706	815	2,988
HIV-negative clients	2,286	4,527	2,286	8,523
HET	1,459	2,933	303	1,131
IDU	245	555	290	1,182
MSM	582	1,040	1,693	6,210
Estimated number of infections associated with the budget cuts (95% CI)	55.0 (19.1, 108.8)	70.5 (23.5, 142.0)	−47.6 (−112.8, −7.9)	−466.1 (−997, −135)
Percent change from the 2,874 annual new diagnoses in pre-cut years	1.91%	2.45%	−1.66%	−16.22%
Expected life-time treatment cost attributable to budget cuts in HIV prevention: $ in million (95% CI)	20.2 (6.0, 42.4)	25.9 (7.3, 55.8)	−17.5 (−44.3, −2.6)	−171.1 (−387, −43.5)
Estimated number of infections associated with the budget cuts by program and transmission category				
Testing and partner services	21.5	45.4	−68.3	−405.4
HET	1.7	2.5	2.8	1.8
IDU	0.9	1.1	0.1	−3.7
MSM	18.9	41.8	−71.2	−403.5
Risk reduction for positive	25.6	19.7	18.5	−39.3
HET	2.7	2.2	4.0	2.7
IDU	0.3	0.2	0.0	−1.4
MSM	22.7	17.3	14.5	−40.7
Risk reduction for negative	7.9	5.4	2.1	−21.4
HET	0.1	0.1	0.2	0.1
IDU	0.2	0.1	0.1	0.0
MSM	7.6	5.2	1.8	−21.5

For scenario 1, we estimated that, based on the relative cost-effectiveness of testing compared with risk reduction programs, the redirection of a greater proportion of prevention funding to testing over risk reduction, compared with programmatic allocations in pre-cut timeframe, averted 15 infections that otherwise would have occurred (Table 4). In other words, had these funds not been reallocated to focus more heavily on testing, the budget cut would have resulted in a 2.45% increase over those otherwise expected. Under scenario 2, if testing and risk reduction were additionally allocated to transmission categories proportionate to each group’s contribution to HIV prevalence in the 15 funded jurisdictions, 46 additional infections could have been averted, for a 1.66% decrease in the total number of new diagnoses in all 59 jurisdictions, compared to the pre-cut timeframe. Under scenario 3, if funding for HIV prevention were restored to the pre-cut budget of $21.8 million and all funds were allocated among testing and risk reduction in the same proportion as in fiscal year 2009–2010, and services were allocated to transmission categories proportionate to each group’s contribution to HIV prevalence in all 59 jurisdictions, 466 new cases (16%) could be averted each year compared to the pre-cut timeframe.

**Figure 1:**
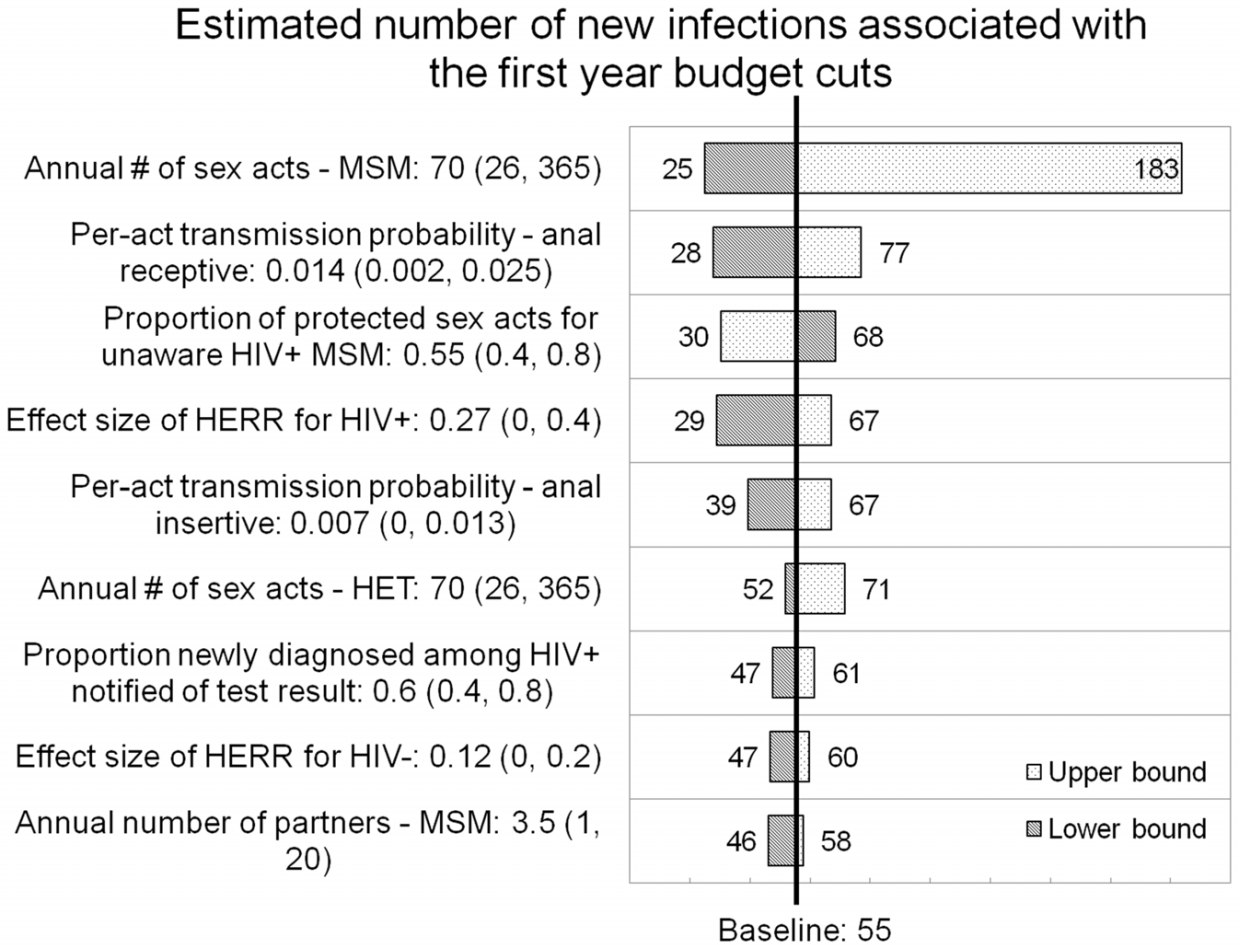
One-way sensitivity analysis. We plotted the input parameters whose change to either the lower or the upper bound resulted in a change of 10% or more in the additional number of new infections associated with the first year of budget cuts. The shadow bar corresponds to the lower bound and the dotted bar corresponds to the upper bound value associated with a particular parameter. For example, if the annual number of sex acts for MSM was 365, the expected number of new infections associated with the first year of the budget cut would increase 236% to 183, from the baseline estimate of 55. If the annual number of sex acts for MSMs was 26, the expected number of new infections associated with the first year budget cuts would decrease by 55% to 25, from the baseline estimate.

We presented the results of one-way sensitivity analysis in a tornado graph (Figure 1). In the one-way sensitivity analysis, the most influential parameter was the annual number of sex acts for MSM. If MSM were assumed to have anal sex every day (or 5 times as frequently as the baseline value of 70), the expected number of new infections associated with the budget cut would increase 2.5 times. If MSM were assumed to have anal sex every two weeks (or a third of the baseline value), the expected number of new infections associated with the budget cut would have decreased by almost 50%. Other influential parameters included the per-act transmission probabilities for anal sex, the proportion of sex acts protected by condoms for undiagnosed positive MSM, the effect size of risk reduction for HIV-infected and uninfected individuals, the annual number of sex acts for HET, the proportion of individuals receiving a first-time positive test result out of all of those who received a positive test result, and the annual number of partners of MSM. Other variables tested changed the number of new infections associated with the budget cut by less than 10%. In the probabilistic sensitivity analysis, when we varied all the parameters together in the base case scenario, the number of infections associated with the budget cut ranged from 19.1 to 108.8, and the associated lifetime treatment costs ranged from $6.1 to $42.4 million.

## Discussion

HIV prevention funding for California’s 59 local health departments outside of Los Angeles and San Francisco declined 70%, by almost $16 million in fiscal year 2009–2010. As a result, an estimated 348 fewer persons with HIV were diagnosed and 8,000 fewer clients were served by risk reduction programs. We estimated that 55 more HIV infections occurred because of the first year of budget cuts, generating $20 million in lifetime treatment costs to the health care system, and indicating that California’s pre-cut HIV prevention funding generated more in medical care savings than the cost of prevention programs.

This study systematically analyzed the effects of HIV prevention programs related to testing and risk reduction on annual transmission and acquisition rates among MSM, HET and IDU. It found that MSM, whose transmission and acquisition rates are highest, also achieve the largest reductions in new HIV cases when they are served by prevention programs. For all transmission categories, the diagnosis of a new infection led to the greatest reduction in annual transmission rates, followed by risk reduction for persons living with HIV. This is because among HIV-infected but undiagnosed individuals, awareness of HIV infection has been shown to increase condom use to a greater extent and for a more sustained period than receiving risk reduction services alone [11]–[13]. Individuals diagnosed with HIV also then have the option to seek treatment to reduce their HIV viral loads, protecting their own health and substantially reducing their ability to transmit the infection to others. Generally, risk reduction for HIV-uninfected persons had modest effects, although it was higher for MSM than for HIV-positive HET and IDU of both genders.

Our findings have been generally supported by other studies [28]–[30]. Holtgrave et al. concluded that promoting knowledge of HIV serostatus for undiagnosed infected persons is critical and that prevention services should focus on HIV-infected individuals [28], [29]. Lasry et al. further underscored the importance of greater focus on HIV-infected MSM and IDU, who are at the highest risk of transmitting the disease [30]. Overall, our analyses and those reported by other studies highlight the benefits of allocating resources to testing, prioritizing risk reduction programs to those who already are infected, and directing HIV prevention programs to MSM.

Findings of the sensitivity analysis indicate that the main outcome, the expected number of new infections associated with the cuts, was sensitive to biological and behavioral parameters for MSM, such as their annual number of sex acts, per-act transmission probabilities of anal sex, proportion of all sex acts protected by condom, and number of partners. Our sensitivity analysis underscores the important role of MSM in the HIV epidemic in California (and many other parts of the United States), and the need for more accurate data on their sexual behaviors. HIV prevalence did not play a key role in this analysis, because when resources were focused on 15 jurisdictions with 87% of the new diagnoses reported in all 59 jurisdictions, the positivity rate among those tested declined slightly. In general, however, HIV prevalence is likely to be an important factor in the cost-effective targeting of prevention resources both because of a theoretically higher positivity rate among those tested and because of a greater likelihood of exposure to HIV among uninfected individuals.

Our analysis is subject to several limitations. Our estimate of the effect of the budget cut does not provide a complete picture for the State of California because the analysis does not consider San Francisco and Los Angeles counties, where half of all Californians living with HIV/AIDS reside. However, the budget and programmatic data for San Francisco and Los Angeles were not sufficiently complete to analyze the impact of the budget cuts in those jurisdictions. We believe our overall findings – that the most efficient allocation of HIV prevention funds in the 59 studied jurisdictions would focus on MSM, with testing prioritized over risk reduction, is likely to hold true for Los Angeles and San Francisco, given that 87% of the living cases of HIV and 81% of new diagnoses in these two counties are among MSM. Our analysis only captures the first generation of transmission by those assumed to become infected with HIV as a consequence of the service reductions, making our estimates conservative. We assumed HIV prevention services provided in California achieve the same level of efficacy reported in published studies. In reality, the delivery and effectiveness of programs likely varies across jurisdictions. Reductions in HIV prevention services, particularly those designed to decrease risky sexual behaviors, could have resulted in increases in other sexually transmitted diseases or unintended pregnancy. The data available to us did not allow us to examine those potential effects.

Most of our analytic scenarios did not take into account possible barriers, including cost, to expanding testing programs or reaching greater numbers of MSM (36% of the MSM at risk for HIV infection). To the extent that these barriers exist, our estimates of new HIV infections averted from budget reallocation are too high. The estimates of annual transmission rates, and reductions in transmission rates associated with prevention services, based on Bernoulli process models, rely on self-reported behavioral data, which are subject to recall and social desirability bias, and other uncertain inputs. Parameter uncertainty is reflected in the results of the probabilistic sensitivity analysis, which provides wide intervals around the base case estimates of new cases associated with the budget cuts and corresponding lifetime HIV treatment costs. The probabilistic sensitivity analysis and the scenario analyses do, however, point to relatively more efficient allocation decisions. Although we did not perform a formal budget optimization analysis, our exploration of various budget scenarios suggest where the California state and local health departments avoided additional increases in HIV infections following the severe budget cut, as well as how additional infections might have been prevented. Our analysis did not examine the impact of the simultaneous state cuts to HIV care and treatment programs, which may as well have resulted in additional infections.

Changes in federal allocation of HIV prevention funding [31], and the lack of data from San Francisco and Los Angeles counties and on state budget cuts to HIV treatment programs, will likely make validation of our impact estimates challenging. However, we recommend that the state Office of AIDS continue to closely monitor trends in new HIV diagnoses and incidence and use these data to better understand the effects of the budget cut and to further refine future funding allocations.

One estimate suggests that achieving National HIV/AIDS Strategy prevention goals by 2015 will require an additional annual investment of $420 million [32]. However, overall funding for HIV prevention has decreased due to state budget cuts in spite of modest increases in federal funding [5], [6]. While the National HIV/AIDS Strategy calls for a 25% decrease in the annual number of new HIV infections by 2015, we estimate that California’s budget cut resulted in a 2% annual increase in new cases in the 59 jurisdictions studied. Restoring state HIV prevention funding to these jurisdictions and allocating resources more strategically to the most cost-effective prevention programs and to populations at highest risk for transmission could have substantial public health benefits and achieve considerable progress toward meeting (but not fully achieving) national goals. Although reductions in funding for HIV prevention have the potential to increase HIV infections, analyses like those described in this paper and other resource allocation tools can help program planners maximize the number of HIV infections prevented given available funding. Important opportunities exist to prevent even more HIV infections and reduce HIV treatment costs by careful allocation of existing HIV prevention funds. Failure to respond to these opportunities in a strategic and timely manner will lead to new HIV infections that could have been prevented, increased health care costs, and an incalculable burden on the lives of the men and women who become infected with HIV, their families and communities.

## Supporting Information

Table S1 **Baseline value, range, and distribution of input parameters for multivariate sensitivity analysis.**
(DOCX)
