# Supplementary material for: Estimating the Impact of State Budget Cuts and Redirection of Prevention Resources on the HIV Epidemic in 59 California Local Health Departments
Source: PLoS One. 2013 Mar 8;8(3):e55713. doi: 10.1371/journal.pone.0055713 (PMC3592871; doi:10.1371/journal.pone.0055713)
Supplement: Table S1 — Baseline value, range, and distribution of input parameters for multivariate sensitivity analysis. (DOCX) [file pone.0055713.s001.docx]

Table S1 Supplement: baseline value, range, and distribution of input parameters for multivariate sensitivity analysis

| Parameter | Value (Range) | Distribution | Source |
| --- | --- | --- | --- |
| HIV prevalence in California |  |  |  |
| HET | 1.0% (0.75–1.25%) | Normal | [[1](#_ENREF_1)] |
| IDU | 5.9% (4.0-10.2%) | Normal | [[2](#_ENREF_2)] |
| MSM | 19.1% (12.8–25.35%) | Normal | [[3](#_ENREF_3)] |
| Proportion of diagnosed HIV-infected persons who are linked to care | 77% (60-85%) | Normal | [[4](#_ENREF_4)] |
| Proportion of HIV-infected persons linked to care who are retained in care | 66% (50-80%) | Normal | [[4](#_ENREF_4)] |
| Proportion of HIV-infected persons, retained in care, who have started ART | 88% (70-90%) | Normal | [[4](#_ENREF_4)] |
| Proportion of HIV-infected persons who started ART who are adherent to ART (i.e., achieve viral load suppression) | 77% (60-85%) | Normal | [[4](#_ENREF_4)] |
| HIV transmission probability per unprotected act |  |  |  |
| Vaginal receptive | 0.08% (0.06–0.11%) | Normal | [[5](#_ENREF_5)] |
| Vaginal insertive | 0.04% (0.01–0.14%) | Normal | [[5](#_ENREF_5)] |
| Anal receptive | 1.40% (0.2–2.5%) | Normal | [[6](#_ENREF_6),[7](#_ENREF_7)] |
| Anal insertive | 0.70% (0-1.3%) | Normal | [[6](#_ENREF_6),[7](#_ENREF_7)] |
| Contaminated needle sharing | 0.30 (0.24%-0.65%) | Normal | [[8](#_ENREF_8)] |
| Annual number of sexual partners |  |  |  |
| HET | 1.21 (1-20) | Lognormal | [[9](#_ENREF_9)] |
| IDU | 3 (1-20) | Lognormal | [[10](#_ENREF_10)] |
| MSM | 3.5 (1-20) | Lognormal | [[11](#_ENREF_11)] |
| Annual number of sex acts (all partners) |  |  | [[12](#_ENREF_12)] |
| HET | 70 (26-365) | Lognormal | [[13-15](#_ENREF_13)] |
| IDU | 70 (26-365) | Lognormal | [[13-15](#_ENREF_13)] |
| MSM | 70 (26-365) | Lognormal | [[13-15](#_ENREF_13)] |
| Annual number of intravenous injections (all partners) | 200 (100-300) | Lognormal | [[16](#_ENREF_16),[17](#_ENREF_17)] |
| Proportion of protected sex acts for undiagnosed HIV-infected individuals and uninfected individuals who do not participate in risk reduction |  |  |  |
| HET | 20% (10-50%) | Normal | [[18](#_ENREF_18)] |
| IDU | 20% (10-50%) | Normal | [[18](#_ENREF_18)] |
| MSM | 55% (40-80%) | Normal | [[19](#_ENREF_19)] |
| Proportion of needle sharing acts among all injections for IDU who do not receive any prevention interventions | 15% (5-25%) | Normal | [[20](#_ENREF_20),[21](#_ENREF_21)] |
| Proportion of new diagnoses among positives notified of test results | 60% (40-80%) | Normal | [[20](#_ENREF_20),[22](#_ENREF_22)] |
| Reduction in unprotected sex acts for aware HIV-infected individuals compared with unaware | 53% (45-60%) | Normal | [[23](#_ENREF_23)] |
| Intervention effect size of HIV education and risk reduction for positive clients | 27% (0-40%) | Normal | [[24-28](#_ENREF_24)] |
| Intervention effect size of HIV education and risk reduction for negative clients | 12% (0-20%) | Normal | [[29-31](#_ENREF_29)] |
| Condom efficacy for per-act transmission | 80% (65–95%) | Normal | [[32](#_ENREF_32)] |
| Reduction in per-act transmissibility for HIV-infected individuals who achieve viral load suppression | 96% (50-100%) | Normal | [[33-37](#_ENREF_33)] |
| Reduction in needle sharing infectivity for infected IDU who achieve viral load suppression | 50% (0-90%) | Normal | [[38](#_ENREF_38)] |
| HIV lifetime treatment cost (2009 $) | 367,134 (184,000-550,000) | Normal | [[39](#_ENREF_39)] |

**Reference**

1. Centers for Disease Control and Prevention (2001) HIV Prevalence Trends in Selected Populations in the United States: Results from National Serosurveillance, 1993–1997. Atlanta, GA: Prevention Services Research Branch, Division of HIV/AIDS Prevention–Surveillance and Epidemiology, National Center for HIV, STD, and TB Prevention, Centers for Disease Control and Prevention.

2. Friedman SR, Lieb S, Tempalski B, Cooper H, Keem M, et al. (2005) HIV among injection drug users in large US metropolitan areas, 1998. J Urban Health 82: 434-445.

3. Xia Q, Osmond DH, Tholandi M, Pollack LM, Zhou W, et al. (2006) HIV prevalence and sexual risk behaviors among men who have sex with men: results from a statewide population-based survey in California. J Acquir Immune Defic Syndr 41: 238-245.

4. Centers for Disease Control and Prevention (2011) Vital Signs: HIV Prevention Through Care and Treatment - United States. MMWR Morb Mortal Wkly Rep 60: 1618-1623.

5. Boily MC, Baggaley RF, Wang L, Masse B, White RG, et al. (2009) Heterosexual risk of HIV-1 infection per sexual act: systematic review and meta-analysis of observational studies. Lancet Infect Dis 9: 118-129.

6. Baggaley RF, White RG, Boily MC (2010) HIV transmission risk through anal intercourse: systematic review, meta-analysis and implications for HIV prevention. Int J Epidemiol 39: 1048-1063.

7. Varghese B, Maher JE, Peterman TA, Branson BM, Steketee RW (2002) Reducing the risk of sexual HIV transmission: quantifying the per-act risk for HIV on the basis of choice of partner, sex act, and condom use. Sex Transm Dis 29: 38-43.

8. Baggaley RF, Boily MC, White RG, Alary M (2006) Risk of HIV-1 transmission for parenteral exposure and blood transfusion: a systematic review and meta-analysis. AIDS 20: 805-812.

9. Chandra A, Mosher WD, Copen C, Sionean C (2011) Sexual behavior, sexual attraction, and sexual identity in the United States: data from the 2006-2008 National Survey of Family Growth. Natl Health Stat Report: 1-36.

10. Metsch L, Zhao W, Lalota M, Beck D, Forrest D, et al. Serosorting Practices among Injection Drug Users (IDUs) in South Florida; 2007 National HIV Prevention Conference; Atlanta, Georgia.

11. Sanchez T, Finlayson T, Drake A, Behel S, Cribbin M, et al. (2006) Human immunodeficiency virus (HIV) risk, prevention, and testing behaviors--United States, National HIV Behavioral Surveillance System: men who have sex with men, November 2003-April 2005. MMWR Surveill Summ 55: 1-16.

12. Long EF, Brandeau ML, Owens DK (2010) The cost-effectiveness and population outcomes of expanded HIV screening and antiretroviral treatment in the United States. Ann Intern Med 153: 778-789.

13. Fortenberry JD, Schick V, Herbenick D, Sanders SA, Dodge B, et al. (2010) Sexual behaviors and condom use at last vaginal intercourse: a national sample of adolescents ages 14 to 17 years. J Sex Med 7 Suppl 5: 305-314.

14. Herbenick D, Reece M, Schick V, Sanders SA, Dodge B, et al. (2010) Sexual behaviors, relationships, and perceived health status among adult women in the United States: results from a national probability sample. J Sex Med 7 Suppl 5: 277-290.

15. Reece M, Herbenick D, Schick V, Sanders SA, Dodge B, et al. (2010) Sexual behaviors, relationships, and perceived health among adult men in the United States: results from a national probability sample. J Sex Med 7 Suppl 5: 291-304.

16. Grigoryan A, Shouse RL, Durant T, Mastro TD, Espinoza L, et al. (2010) HIV Infection Among Injection-Drug Users-34 States, 2004-2007 (Reprinted from MMWR, vol 58, pg 1291-1295, 2009). Jama-Journal of the American Medical Association 303: 126-128.

17. Lansky A, Drake A, Pham HT (2009) HIV-Associated Behaviors Among Injecting-Drug Users-23 Cities, United States, May 2005-February 2006 (Reprinted from MMWR, vol 58, pg 329-332, 2009). Jama-Journal of the American Medical Association 302: 376-377.

18. Reece M, Herbenick D, Schick V, Sanders SA, Dodge B, et al. (2010) Condom use rates in a national probability sample of males and females ages 14 to 94 in the United States. J Sex Med 7 Suppl 5: 266-276.

19. Finlayson TJ, Le B, Smith A, Bowles K, Cribbin M, et al. (2011) HIV risk, prevention, and testing behaviors among men who have sex with men--National HIV Behavioral Surveillance System, 21 U.S. cities, United States, 2008. MMWR Surveill Summ 60: 1-34.

20. Centers for Disease Control and Prevention (2011) Results of the Expanded HIV Testing Initiative--25 jurisdictions, United States, 2007-2010. MMWR Morb Mortal Wkly Rep 60: 805-810.

21. Centers for Disease Control and Prevention (2011) HIV Testing at CDC-Funded Sites, United States, Puerto Rico, and the U.S. Virgin Islands, 2008-2009. In: Prevention CfDCa, editor. Atlanta, GA: Department of Health and Human Services, Centers for Disease Control and Prevention.

22. Hanna DB, Tsoi BW, Begier EM (2009) Most positive HIV western blot tests do not diagnose new cases in New York City: implications for HIV testing programs. J Acquir Immune Defic Syndr 51: 609-614.

23. Marks G, Crepaz N, Senterfitt JW, Janssen RS (2005) Meta-analysis of high-risk sexual behavior in persons aware and unaware they are infected with HIV in the United States: implications for HIV prevention programs. J Acquir Immune Defic Syndr 39: 446-453.

24. Healthy Living Project Team (2007) Effects of a behavioral intervention to reduce risk of transmission among people living with HIV: the healthy living project randomized controlled study. J Acquir Immune Defic Syndr 44: 213-221.

25. Fisher JD, Fisher WA, Cornman DH, Amico RK, Bryan A, et al. (2006) Clinician-delivered intervention during routine clinical care reduces unprotected sexual behavior among HIV-infected patients. J Acquir Immune Defic Syndr 41: 44-52.

26. Kalichman SC, Rompa D, Cage M, DiFonzo K, Simpson D, et al. (2001) Effectiveness of an intervention to reduce HIV transmission risks in HIV-positive people. Am J Prev Med 21: 84-92.

27. Rotheram-Borus MJ, Swendeman D, Comulada WS, Weiss RE, Lee M, et al. (2004) Prevention for substance-using HIV-positive young people: telephone and in-person delivery. J Acquir Immune Defic Syndr 37 Suppl 2: S68-77.

28. Wingood GM, DiClemente RJ, Mikhail I, Lang DL, McCree DH, et al. (2004) A randomized controlled trial to reduce HIV transmission risk behaviors and sexually transmitted diseases among women living with HIV: The WiLLOW Program. J Acquir Immune Defic Syndr 37 Suppl 2: S58-67.

29. Crosby R, Diclemente RJ, Yarber WL (2009) Correlates of Correct Condom Use Among High-Risk African American Men Attending an Urban STD Clinic in the South. Int J Sex Health 21: 183-191.

30. Jemmott LS, Jemmott JB, 3rd, O'Leary A (2007) Effects on sexual risk behavior and STD rate of brief HIV/STD prevention interventions for African American women in primary care settings. Am J Public Health 97: 1034-1040.

31. Scholes D, McBride CM, Grothaus L, Civic D, Ichikawa LE, et al. (2003) A tailored minimal self-help intervention to promote condom use in young women: results from a randomized trial. AIDS 17: 1547-1556.

32. Weller S, Davis K (2002) Condom effectiveness in reducing heterosexual HIV transmission. Cochrane Database Syst Rev: CD003255.

33. Bunnell R, Ekwaru JP, Solberg P, Wamai N, Bikaako-Kajura W, et al. (2006) Changes in sexual behavior and risk of HIV transmission after antiretroviral therapy and prevention interventions in rural Uganda. AIDS 20: 85-92.

34. Del Romero J, Castilla J, Hernando V, Rodriguez C, Garcia S (2010) Combined antiretroviral treatment and heterosexual transmission of HIV-1: cross sectional and prospective cohort study. BMJ 340: c2205.

35. Donnell D, Baeten JM, Kiarie J, Thomas KK, Stevens W, et al. (2010) Heterosexual HIV-1 transmission after initiation of antiretroviral therapy: a prospective cohort analysis. Lancet 375: 2092-2098.

36. Attia S, Egger M, Muller M, Zwahlen M, Low N (2009) Sexual transmission of HIV according to viral load and antiretroviral therapy: systematic review and meta-analysis. Aids 23: 1397-1404.

37. Cohen MS, Chen YQ, McCauley M, Gamble T, Hosseinipour MC, et al. (2011) Prevention of HIV-1 infection with early antiretroviral therapy. N Engl J Med 365: 493-505.

38. Sanders GD, Bayoumi AM, Sundaram V, Bilir SP, Neukermans CP, et al. (2005) Cost-effectiveness of screening for HIV in the era of highly active antiretroviral therapy. N Engl J Med 352: 570-585.

39. Schackman BR, Gebo KA, Walensky RP, Losina E, Muccio T, et al. (2006) The lifetime cost of current human immunodeficiency virus care in the United States. Med Care 44: 990-997.
